# Domestic

**Published:** 1841-06-12

**Authors:** 


### DOMESTIC.

*Cases of Ligature of the Femoral and the External Iliac Arteries, by* Placide Portal, *Professor of Surgery and Obstetrics, in the Royal University of Palermo.*—Two men lately came to me, afflicted with lues venerea, of a year's standing, one of whom had an aneurism in his ham, the other was laboring under a hæmorrhage in the third superior, and sometimes in the femoral artery, a little lower down. The former had the artery tied, and though the aneurismal tumor was of the enormous size of forty-two inches in circumference, he entirely recovered. The other, whose external iliac artery I tied, though not on account of aneurism, died of consequent hæmorrhages, as these could not be stopped nor checked by any treatment. But the former, his previous venereal taint being now almost eradicated, was treated as a healthy man. In the other, whose very habit of body augured ill, (the operation of tying having become indispensable, as his femoral artery was corroded by phagedenic ulcers,) gangrene and ulceration came on, which destroyed the whole system, as well as the part affected by the operation. Still, on a personal inspection of the body, enough was discovered to show how much nature favors our safety. For the artery was found to have been for the most part obliterated, so as at least to be able to resist the impulse of the blood. Before, therefore, we proceed to tie the artery, we must carefully consider the case, and be sure that those diseases and obstacles which indicate an unfavorable result, may be removed, or at least so far brought under control as to afford a probability that the operation may be attended with success. In men, especially, who have been afflicted with the venereal, all these things ought to be attended to, as experience has sufficiently convinced me that aneurisms of this kind frequently originate from that disease.*

Case 1st.—Cajetanus Basile, a native of the island of Lipari, aged 29 years, and a gunner in the royal navy, of lymphatic temperament and moderate firmness of body, after being repeatedly affected with the venereal, was admitted into the hospital of St. Francis Xavier for the treatment of a tumor in the ham, which had appeared about a month before. He had previously been afflicted with buboes, venereal ulcers, blenorrhagia, and pains in the bones. These were treated by gentle remedies, and it was also hoped that the tumor might, through these means, be discussed. A linament composed of almond oil, opium and camphor, was applied to it. But it proved useless, and as the swelling rapidly increased, he sought admission into the hospital. Here he was treated with oxygenated ointment for the venereal affection, as the cause of his ailments, and for the two first days the pains were mitigated. But on the next, they returned with increased violence in the ham, the tumor enlarged in size and pulsated. Emollient cataplasms, cold applications, the extract of belladonna, and laurel water, all failed to relieve, and he was finally removed from the medical to the surgical ward of the hospital.

The surgeon in attendance unfortunately applied compression with cloths dipped in decoction of oak bark. The result was most disastrous. The pain became more severe, and the inflammation extended, so as to threaten, by its livid appearance, the approach of gangrene.—The tumor also had now enlarged to the enormous size of forty-two inches in circumference. While in this imminent danger, I was invited by General Carascosa to visit the patient. On removing the bandages and viewing the condition of the parts, but one remedy remained—to tie the artery; and in this most of the professors of the hospital concurred.

An incision of three inches was made along the margin of the sartorius, in the triangular space, so that I could reach the aponeurosis.—This was then opened, and with a probe pointed bistoury I brought forward the sheath, and cut into it. Proceeding with the operation, a ligature composed of three linen threads waxed, was introduced by means of an eyed probe from beneath and outwards. On tying it, the pulsation of the popliteal in the tumor ceased. I then proceeded to apply the second ligature, and having brought the ends of the thread to the upper mouth of the wound, I dressed it with lint, covered with the cerate of Galen. The wound was then closed with ligatures, and the patient was returned to his bed. Absolute quiet, a low diet and cold fomentations were prescribed, with sweetened lemonade as a drink.

The patient suffered extreme pain in less than an hour after the operation, both in the wound and the corresponding part of the thigh. He presently however fell asleep, and while in this state, the artery near the ligature was observed to beat very strongly, the pulse also rose, and fever was evidently setting in. Bleeding to eight ounces was prescribed, followed by cold fomentations. This reduced the arterial action. At evening the extremities became quite cold, and warm cloths were applied to restore the temperature.

On the next day the heat of the body was natural, the tongue moist, but the bowels were constipated. A dose of castor oil was rejected by vomiting, but the exhibition of sweetened cream of tartar in solution produced copious discharges from the bladder and the intestines. Towards evening the pulse became quick and

* As to the methods of tying the arteries, there has always appeared to me to be great danger in drawing the ligature too tight, since from this, ulceration of the artery and consequent hæmorrhage generally proceed. And this I think takes place in consequence of the interruption of the circulation, by which the part compressed ought to be nourished. In order to avoid danger of this kind, I use the double knot only for security, so that the parts may be brought together without tight constriction, and may thus be preserved from ulceration by a free circulation. That I am not deceived in this reasoning, daily experience in brute animals, and in men afflicted with aneurism and lesion of the artery, sufficiently shows. Simple constriction is in my judgment the only proper method of tying the arteries.

hard, with increased fever. Venesection was again resorted to, with tartarized drinks, (*portionibus tartaro saturis.*) These relieved the symptoms, and the patient slept five hours during the night. On the 3d, 4th, and 5th days, there was still fever, with pain in the diseased part, but laxative medicines produced copious discharges and the unfavorable appearances abated.

On the 6th, the tent was removed; it was saturated with a bloody sanies; a new one was inserted; the wound now extended to the lower extremity. During the 7th and 8th days bleeding was for the third time employed, and an infusion of digitalis was prescribed. Symptoms greatly mitigated.

9th. On removing the tent it was found tinged with blood derived from the margin of the wound, and not from the artery. Another one was applied.

10th. The wound is cicatrizing, but the febrile symptoms are again high; they are reduced by another bleeding.

11th and 12th. Symptoms as before.

13th. The patient has been shifted to another bed. On applying a fresh tent, the tumor is found filled with black and very fœtid bloody clots. To be anointed with ointment of Storax, and an antiseptic cataplasm to be applied.

14th. Pus is formed in the tumor, and its size is diminishing. External applications as yesterday, with chicken broth and acidulated drinks.

15th. The ligature has come away. A large quantity of sanious fluid has been discharged from the tumor. A bandage is brought round it, and the strength supported by chicken broth.

16th to 18th. The incision being now nearly healed, was touched in its centre with lunar caustic. The tumour is diminishing. To take ass's milk and Iceland moss.

19th and 20th. The tumor discharges tolerably good pus. A dark spot has appeared on the small toe of the right side. To apply an antiseptic cataplasm. By the 22d day a scab was thrown off.

23d and 24th. Three sores have appeared; on the upper and outer side of the right foot, another on the outer ankle, and the third on the lower part of the leg. To be treated with ointment of thorax, pulvis chinæ and camphor, and the antiseptic cataplasm.

25th. The sores appear tending to gangrene. A disarticulation of the small toe has occurred spontaneously. To continue the cataplasm, and to use ass's milk and Iceland moss.

26th. A new ulcer near the heel, at the internal margin of the foot.

27th and 28th. The wound caused by the incision is now healed, but the interior of the tumor is filled with proud flesh, (carnosis globulis.) The ulcers on the leg and foot are deeper and larger in size. Neither general or topical remedies seem to check their progress. They are accompanied with most severe pain and swelling of the extremity, and appear to be tending to gangrene. The necessity of amputation was canvassed, and it was agreed to perform it on the next day.

30th and 31st. The amputation has been delayed, as the symptoms are somewhat mitigated.

35th. In consequence of the inconsiderate use of aqua phagedenica, the ulcers appeared worse. To omit the use of all mercurial remedies, and to apply only the cerate of Galen.

38th. The ulcers have been treated with the cautery, and all the apparently gangrenous portions having been removed, the whole took on a healthy appearance. Granulations occurred on the next day, and the general tendency was toward healing.

On the 27th of October, and the 40th day after the operation, the patient was cured of all ulcers, except one at the heel, caused by a caries of the os calcis. This also was treated with the cautery, and he finally left the hospital perfectly recovered.

Case 2. Antonius Bevilacqua, of Palermo, aged 30, a servant, of good constitution, but dissolute in his habits, had been suffering with buboes, for which he obtained admissiion into the hospital. Mercurial friction and diaphoretics were prescribed, with some advantage, and the bubo in the left groin was sensibly diminished. But that in the right proceeded to the state of suppuration, and he was transferred to the surgical side. Here it was treated with an emollient cataplasm, and when a fluctuation was perceived, it was opened, and discharged a copious sanies. The wound was then dressed with digestive ointment.

Its appearance, however, became more and more unfavourable, and a phagedenic wash was applied, but without any benefit. The China root in decoction, as also in powder, to which opium and camphor were added, was next exhibited as the most approved antiseptic; but all proved of no avail, and the house surgeon, under an idea that the presence of the inguinal glands might prevent cicatrization, removed them. The wound was then dressed with lint dipped in ointment of storax.

On the next day, while at stool, the patient felt a moisture in the groin, and on examination found blood flowing from it. He made pressure on the part, and the surgeon, on being summoned, applied a compress, which restrained the hæmorrhage for the time.

On consultation, the necessity of tying the external iliac was urged, but this was doubted by some, until a second and more copious hæmorrhage settled all doubts. It was evident from the exhausted condition of the patient, that he could not survive a third discharge of blood.

An incision of three inches was made from the inferior convexity of the anterior iliac spine to the symphisis pubis. This divided the skin and the adipose substance. The superficial fascia, and the obliquus internus were next divided, the spermatic cord was laid bare, and by means of the finger and the bistoury, I made an opening into the transverse fascia, and thus reached the inguinal canal. The artery was separated, and tied in two places. The extremities of the ligature were brought to the upper part of the wound, which was dressed as in the former case, and a graduated compress brought around the limb.

The patient, both during the day of the operation and the subsequent one, fell into a state of stupor and extreme weakness, the pulse was low, and the heat of the body below the natural standard. Nutritive drinks were administered, and external heat applied to the limb. The symptoms, however, continued to be unfavourable; a bloody discharge passed from the wound, and although the circulation was maintained in the crural artery by the epigastric, yet fungous appearances and a gangrenous tendency manifested themselves; and this gradually extended, accompanied with all the symptoms of sinking, until death occurred.

On examination, in the presence of the hospital physicians, the ligature was found in place one inch above the origin of the two collaterals of the external iliac. The sides of the artery adhered, and below it was a cutaneous thrombus, impervious and adhering to the artery. The viscera of the abdomen bore the marks either of inflammation or of commencing gangrene. The artery, from the ligature to the heart, was healthy. The thoracic viscera and the brain were sound.—*Trans. Med. Society of New York.*

